# Raloxifene inhibits tumor growth and lymph node metastasis in a xenograft model of metastatic mammary cancer

**DOI:** 10.1186/1471-2407-10-566

**Published:** 2010-10-19

**Authors:** Masa-Aki Shibata, Junji Morimoto, Eiko Shibata, Hitomi Kurose, Kanako Akamatsu, Zhong-Lian Li, Moriaki Kusakabe, Masahide Ohmichi, Yoshinori Otsuki

**Affiliations:** 1 Department of Anatomy and Cell Biology, Division of Life Sciences, Osaka Medical College, 2-7 Daigaku-machi, Takatsuki, Osaka 569-8686, Japan; 2 Laboratory Animal Center, Osaka Medical College, 2-7 Daigaku-machi, Takatsuki, Osaka 569-8686, Japan; 3 Department of Systems Bioscience for Drug Discovery, Graduate School of Pharmaceutical Sciences, Kyoto University, Kyoto, Japan; 4 Research Center for Food Safety, University of Tokyo Graduate School of Agricultural and Life Sciences, Tokyo, Japan; 5 Department of Gynecology, Osaka Medical College, 2-7 Daigaku-machi, Takatsuki, Osaka 569-8686, Japan; 6 Department of Bioscience, National Cardiovascular Center Research Institute, Suita, Osaka, Japan

## Abstract

**Background:**

The effects of raloxifene, a novel selective estrogen receptor modulator, were studied in a mouse metastatic mammary cancer model expressing cytoplasmic ERα.

**Methods:**

Mammary tumors, induced by inoculation of syngeneic BALB/c mice with BJMC3879luc2 cells, were subsequently treated with raloxifene at 0, 18 and 27 mg/kg/day using mini-osmotic pumps.

**Results:**

*In vitro *study demonstrated that the ERα in BJMC3879luc2 cells was smaller (between 50 and 64 kDa) than the normal-sized ERα (66 kDa) and showed cytoplasmic localization. A statistically significant but weak estradiol response was observed in this cell line. When BJMC3879luc2 tumors were implanted into mice, the ERα mRNA levels were significantly higher in females than in males. *In vitro *studies showed that raloxifene induced mitochondria-mediated apoptosis and cell-cycle arrest in the G1-phase and a decrease in the cell population in the S-phase. In animal experiments, tumor volumes were significantly suppressed in the raloxifene-treated groups. The multiplicity of lymph node metastasis was significantly decreased in the 27 mg/kg group. Levels of apoptosis were significantly increased in the raloxifene-treated groups, whereas the levels of DNA synthesis were significantly decreased in these groups. No differences in microvessel density in tumors were observed between the control and raloxifene-treated groups. The numbers of dilated lymphatic vessels containing intraluminal tumor cells were significantly reduced in mammary tumors in the raloxifene-treated groups. The levels of ERα mRNA in mammary tumors tended to be decreased in the raloxifene-treated groups.

**Conclusion:**

These results suggest that the antimetastatic activity of raloxifene in mammary cancer expressing cytoplasmic ERα may be a crucial finding with clinical applications and that raloxifene may be useful as an adjuvant therapy and for the chemoprevention of breast cancer development.

## Background

The selective estrogen receptor modulators (SERMs) exhibit specific estrogen-receptor (ER) agonistic and antagonistic activity by binding to ERα and/or β. Of the SERMs, tamoxifen and raloxifene differ from estrogens in that they exert both agonistic and antagonistic properties. Tamoxifen acts as an antagonist in the breast and an agonist in the bone and uterus. Therefore, tamoxifen is used clinically as a therapeutic agent to treat ER-positive breast cancer. Although tamoxifen prevents ER-positive breast cancers [1], it increases the incidence of endometrial cancer [2,3]. Raloxifene has antiestrogenic effects on the breast and bone, but it does not have an estrogenic effect on the uterus. These SERMs have different biological actions because raloxifene recruits a co-repressor in endometrial carcinoma cells, whereas tamoxifen induces a co-activator [4]. In fact, raloxifene inhibits carcinogen-induced mammary carcinoma [5-7] and colon carcinoma [8] in animal models.

The Study of Tamoxifen and Raloxifene (STAR) trial has shown that raloxifene is as effective as tamoxifen in reducing the risk of invasive breast cancer, and there were less cases of endometrial cancer with raloxifene than with tamoxifen [9]. Results of other clinical trials of raloxifene, such as the Multiple Outcomes of Raloxifene Evaluation (MORE) [10], Continuing Outcomes Relevant to Evista (CORE) [11] and the Raloxifene Use for The Heart (RUTH) [12] trials, showed that raloxifene reduces the risk of invasive ER-positive breast cancer in postmenopausal women. As compared with tamoxifen, raloxifene appears to have fewer serious side effects, including endometrial cancer, venous thrombosis and cataracts, without compromising the breast cancer chemoprevention strategy [13].

Breast cancer is the most common malignancy in women worldwide and is one of the most lethal carcinomas. In Japan, the incidence of breast cancer is continuously increasing and the disease now ranks fifth as a cause of female mortality; the number of breast cancer deaths in Japan increased 2.6-fold between 1975 and 1998 [14]. The lethality of breast cancer is largely due to metastasis; the most common sites are lung, lymph nodes, liver, and bone. Effective and less toxic chemopreventive agents are needed to delay the progression of breast cancer and prolong life.

Here, we investigated the chemopreventive ability of raloxifene, especially its antimetastatic ability, in a mouse metastatic mammary cancer model expressing cytoplasmic ERα. This mammary cancer model has a p53 mutation that shows a metastatic spectrum similar to that seen in human breast cancers [15-17]. In addition, we studied the apoptosis pathway, DNA synthesis, and cell cycle in metastatic mouse mammary carcinoma cells treated with raloxifene *in vitro*.

## Methods

### Experimental regimen

Raloxifene hydrochloride was purchased from Sigma Co. (St. Louis, MO, USA). For *in vitro *use, raloxifene was dissolved in dimethylsulfoxide (DMSO), and aliquots of 20 mM stock solution were stored at -20°C.

### Cell line and animals

The BJMC3879luc2 mammary carcinoma cell line [18] was generated by stable transfection of luc2 (an improved *firefly luciferase *gene) into parent cell line BJMC3879. The mammary tumors arising from BJMC3879 cell implantation had a high propensity for metastasis into the lymph nodes and lungs [15-17], a trait retained through culture. BJMC3879luc2 cells were maintained in RPMI 1640 medium containing 10% fetal bovine serum with streptomycin/penicillin in an incubator under 5% CO_2_.

Thirty female 6-week-old BALB/c mice were used in this study (Japan SLC, Hamamatsu, Japan). The animals were housed five per plastic cage on wood chip bedding with free access to water and food under controlled temperature (21 ± 2°C), humidity (50 ± 10%), and lighting (12-12 h light-dark cycle). All animals were held for a 1-week acclimatization period before study commencement. Mice were treated in accordance with the procedures outlined in the Guide for the Care and Use of Laboratory Animals in Osaka Medical College, the Japanese Government Animal Protection and Management Law (No. 105) and the Japanese Government Notification on Feeding and Safekeeping of Animals (No. 6).

### Estrogen receptor expression

#### Immunofluorescence staining

BJMC3879luc2 cells were grown in 2-well chamber slides and fixed in 4% formaldehyde solution in phosphate buffer. Immunofluorescence staining was performed with anti-ERα rabbit polyclonal antibody (clone MC-20; Santa Cruz Biotechnology, Santa Cruz, CA, USA).

#### Western blotting

Total protein was extracted from whole cell lysates of BJMC3879luc2 cells. Total protein (40 μg) was electrophoretically separated in 14% Tris-glycine gels under reducing conditions and transferred to nitrocellulose membranes. The membrane was incubated with anti-ERα (Santa Cruz Biotechnology) or anti-ERβ (Affinity Bioreagents, Golden, CO, USA) rabbit polyclonal antibodies, followed by secondary antibodies conjugated to HRP. Then, the bound antibody was visualized with enhanced chemiluminescence reagent (Perkin Elmer Life Sciences Inc., Boston, MA, USA). Blots were visualized using a LAS-3000 image analyzer (Fujifilm, Co., Tokyo, Japan). Anti-Bid and anti-actin goat polyclonal antibodies (Santa Cruz Biotechnology) were used as primary antibodies.

#### ERα expression of BJMC3879luc2-implanted tumors in females and males

BJMC3879luc2 cells (5 × 10^6 ^cells/0.3 ml in PBS) were inoculated subcutaneously into the right inguinal mammary fat pad of 10 BALB/c mice (5 females and 5 males). From two to four weeks after the inoculation, the mammary tumors were measured with digital calipers. The tumor volumes were calculated with the following formula: maximum diameter × (minimum diameter)^2 ^× 0.4 [19]. Four weeks after inoculation, mammary tumors were immediately excised under isoflurane anesthesia. Total RNA was extracted, and transcriptional levels of ERα were measured in mammary tumors using real-time reverse transcriptase (RT)-PCR (see "ERα expression in mammary tumors" for details).

### Cell viability

BJMC3879luc2 cells were grown in RPMI-1640 medium supplemented with 10% (v/v) heat-inactivated fetal bovine serum and 2 mM L-glutamine under an atmosphere of 95% air and 5% CO_2 _at 37°C. BJMC3879luc2 cells were plated onto 96-well plates (1 × 10^4 ^cells/well) 1 day before raloxifene treatment. They were subsequently incubated for 24 h with culture medium containing vehicle (DMSO) alone or with medium containing raloxifene at different concentrations up to 80 μM. Cell viability was determined using a CellTiter-Blue Cell Viability Assay (Promega Co., Madison, WI, USA). In addition, to examine the response to estrogen in BJMC3879luc2 cells, one week before the experiment, the medium was changed to a phenol red-free form of RPMI-1640 containing charcoal-stripped fetal bovine serum (steroid-free medium). The cells were seeded at 5 × 10^4 ^cells/well in the culture medium (steroid-free and phenol red-free). After overnight culture, the cells were exposed to 17-β estradiol (E2) at final concentrations of 10^-12 ^to 10^-4 ^M (1 pmole to 100 μmole) for 24 h, and then cell viability was determined as described above.

### TUNEL assay, caspase activity and DNA synthesis

BJMC3879luc2 cells were grown in 2-well chamber slides and treated with 20 μM raloxifene for 48 h. Then, the cells were fixed in 4% formaldehyde solution in phosphate buffer, and terminal deoxynucleotidyl transferase-mediated dUTP-FITC nick end-labeling (TUNEL) staining was performed according to the manufacturer's protocol (Wako Pure Chemical Industries, Osaka, Japan).

BJMC3879luc2 cells were plated onto 96-well plates (1 × 10^4 ^cells/well) 1 day before raloxifene treatment. Cells were treated with 20 μM raloxifene or vehicle alone for 48 and 72 h, and then cell viability was measured using a CellTiter-Blue Cell Viability Assay (Promega). The activities of caspase-8, caspase-9 and caspase-3 were measured using a luminescent assay kit (Promega). Caspase activity was measured in terms of the luminescent signal produced by caspase cleavage of the corresponding substrate using a Luminoskan Ascent device (Thermo Electron Co., Helsinki, Finland). Caspase activity levels were corrected by the corresponding cell viabilities. In addition, cells from the cultures were incubated for 1 h in medium containing 50 μM 5-bromo-2'-deoxyuridine (BrdU), and DNA synthesis of the cells was measured by BrdU incorporation (Cell Proliferation on ELISA, BrdU Chemiluminescence; Roche Diagnostics, GmbH, Mannheim, Germany). Data were also corrected by the corresponding cell viabilities.

### Release of cytochrome c

After incubation in culture medium with or without 20 μM raloxifene for 48 h, both floating and attached cells were harvested, rinsed once in PBS, re-suspended in cell lysis buffer, incubated for 1 h at room temperature, and centrifuged at 1000 × g for 15 min. The resultant supernatant was diluted at least 5-fold. Supernatants containing the cytosolic fraction were collected separately, and the protein concentrations were determined. To determine the cytochrome *c *release into the cytosol, cytochrome *c *was measured using a cytochrome *c *kit (R&D Systems, Inc, Minneapolis, MN, USA).

### Caspase inhibitor experiment

Cells were treated with 10 μM and 100 μM of the following caspase inhibitors for 48 h: z-VAD-fmk against broad-spectrum caspases, Ac-DNLD-CHO against caspase-3, z-IETD-fmk against caspase-8 and z-LEHD-fmk against caspase-9. The caspase inhibitors, with the exception of caspase-3 inhibitor (Peptide Institute, Inc., Osaka, Japan), were purchased from MBL Inc. (Nagoya, Japan). Although DEVD has been generally used as a caspase-3 inhibitor, this sequence has been reported to be non-specific to caspase-3; therefore, Ac-DNLD-CHO was used in the present experiment [20,21]. Two hours after treatment with caspase inhibitors, cells were exposed to 20 μM raloxifene. Cell viability was measured using a fluorescent assay kit (CellTiter-Blue Cell Viability Assay, Promega), and then the activities of caspase-3, caspase-8 and caspase-9 were measured using a luminescent assay kit (Promega). The caspase activity data was then adjusted to account for the corresponding cell viability as previously reported [22].

### Cell-cycle distribution

Flow cytometric analysis was conducted on trypsinized BJMC3879luc2 cell suspensions that were harvested after a 48-h treatment with 20 μM raloxifene and fixed in cold 70% ethanol. The cells were stained with a 50 μg/ml propidium iodide solution containing 100 μg/ml RNase A for 30 min at 37°C and then placed on ice just prior to flow cytometric analysis (EPICS Elite ESP; Coulter Co., Miami, FL, USA). The percentage of cells in each phase of the cell cycle was determined using a Multicycle Cell Cycle Analysis program (Coulter Co.).

### In vivo study of raloxifene in a metastatic mammary cancer model

Two dosages of raloxifene for mice (27 mg/kg and 18 mg/kg) were selected based on the results of other studies [23]. Raloxifene was continuously administered via subcutaneously implanted mini-osmotic pumps (Alzet model 2002, Durect Co., Cupertino, CA, USA) that were calibrated to release 0.5 μl of solution per hour. Raloxifene solutions (47.5 mg/ml and 31.7 mg/ml) in DMSO and 100% ethanol (1:3, v/v) were prepared. Since the pumps were calibrated to release for 14 days, they were replaced every other week.

BJMC3879luc2 cells (5 × 10^6 ^cells/0.3 ml in PBS) were subcutaneously inoculated into the right inguinal mammary fat pad of 30 female BALB/c mice. Two weeks later, when tumors had reached approximately 0.6 cm in diameter, mini-osmotic pumps were used to administer 0, 18 or 27 mg/kg raloxifene for 6 weeks. Individual body weights were recorded weekly. Each mammary tumor was also measured weekly using digital calipers, and tumor volumes were calculated according to the formula of maximum diameter × (minimum diameter)^2 ^× 0.4 [19]. All animals received 50 mg/kg BrdU (Sigma Co.) i.p. at 1 h prior to sacrifice. All surviving mice were euthanized with isoflurane anesthesia at week 6.

### Bioluminescence imaging in vivo

At week 6, five mice in each group were anesthetized by isoflurane inhalation with an SBH Scientific anesthesia system (SBH Designs Inc., Ontario, Canada). Each anesthetized mouse received an intraperitoneal injection of 3 mg of D-luciferin potassium salts (Wako Pure Chemical Industries). Bioluminescence imaging with a Photon Imager (Biospace Lab, Paris, France) was performed. The bioluminescent signals received during the 6-min acquisition time were quantified using Photovision software (Biospace Lab).

### Histopathological analyses

At necropsy, tumors and lymph nodes were removed, fixed in 10% formaldehyde solution in phosphate buffer and processed through to paraffin embedding. The lymph nodes from the axillary and femoral regions were routinely removed, along with lymph nodes that appeared abnormal. In several cases, the uterus was also excised and preserved in fixative solution. Lungs were inflated with formaldehyde solution prior to excision and immersion in fixative; the individual lobes were subsequently removed from the bronchial tree and examined for metastatic foci and similarly processed through to paraffin embedding. All paraffin-embedded tissues were cut into 4-μm-thick sections. Sequential sections were stained with hematoxylin and eosin for histopathological examination or remained unstained for immunohistochemical analysis.

### p53 immunohistochemistry

The labeled streptavidin-biotin (LSAB) method (Dako, Glostrup, Denmark) was used for p53 immunohistochemistry. Unstained sections were immersed in distilled water and heated for antigen retrieval prior to incubation with a p53 mouse monoclonal antibody (Clone Pab240, Santa Cruz Biotechnology) that reacts to the mutant protein in fixed specimens.

### Apoptosis and caspase in mammary tumors

For the quantitative analyses of cell death, sections from paraffin-embedded tumors were assayed using the TUNEL method in conjunction with an apoptosis *in situ *detection kit (Wako Pure Chemical Industries) with minor modifications to the manufacturer's protocol. TUNEL-positive cells (mainly regarded as apoptotic cells) were counted in viable regions peripheral to areas of necrosis in tumor sections. The slides were scanned at low-power (×100) magnification to identify those areas having the highest number of TUNEL-positive cells. Four areas neighboring the highest area of TUNEL-positive cells were then selected and counted at higher (×200-400) magnification. The numbers of TUNEL-positive cells were expressed as numbers per cm^2^.

Active caspase expression of the mammary tumor tissues was immunohistochemically detected using anti-cleaved caspase-3 and cleaved caspase-9 rabbit polyclonal antibodies (Cell Signaling Technology, Danvers, MA, USA). Immunohistochemistry was conducted using the LSAB method, and CSA II amplification (Dako) was additionally applied to detect cleaved caspase-9.

### DNA synthesis in mammary tumors

The tumors from five animals from each treatment group were subsequently evaluated for DNA synthesis rates as inferred by BrdU incorporation. DNA was denatured *in situ *by incubating unstained paraffin-embedded tissue sections in 4 N HCl solution for 20 min at 37°C. The incorporated BrdU was visualized after exposure to an anti-BrdU mouse monoclonal antibody (Clone Bu20a, Dako). The numbers of BrdU-positive S-phase cells per 250 mm^2 ^were counted in four random high-power (×400) fields of viable tissue, and the BrdU labeling indices were expressed as numbers per cm^2^.

### Lymphatic and blood microvascular densities in mammary tumors

To quantitatively assess lymphatic and blood microvessel density in the primary mammary carcinomas, immunohistochemistry based on the LSAB method (Dako) was performed. A hamster anti-podoplanin monoclonal antibody (AngioBio Co., Del Mar, CA, USA) against a lymphatic endothelium marker and a rabbit polyclonal antibody against CD31 (Lab Vision Co., Fremont, CA, USA), a specific marker for blood vessel endothelium, were used. The number of podoplanin-positive lymphatic vessels containing intraluminal tumor cells was also counted. In addition, the number of CD31-positive blood microvessels was counted as previously described [24]. Briefly, the slides were scanned at low-power (×100) magnification to identify those areas having the highest number of vessels. The five areas of highest microvascular density were then selected and counted at higher (×200-400) magnification.

### ERα expression in mammary tumors

Immunohistochemical staining for ERα (anti-ERα rabbit polyclonal antibody, Santa Cruz Biotechnology) was performed using the LSAB method in combination with a CSA II amplification kit (Dako). In addition, the levels of ERα mRNA in mammary tumor tissues were also measured using a real-time reverse transcriptase-polymerase chain reaction (RT-PCR). Total RNA was isolated from two 4-μm sections of each paraffin-embedded tumor using an RNeasy FFPE kit (Qiagen, GmbH, Hilden, Germany), and cDNAs were synthesized according to the manufacturer's instructions (Roche Diagnostics). cDNAs were then amplified using a LightCycler and LightCycler FastStart DNA Master SYBR Green I according to the manufacturer's instructions (Roche Diagnostics). The primer sequences for mouse ERα were 5'-AAAGCTGGCCTGACTCTG-3' and 5'-GATGCTCCATGCCTTTGT-3'. The primer sequences for mouse glyceraldehyde-3-phosphate dehydrogenase (GAPDH), which was used as an internal control, were 5'-TGGCCTTCCGTGTTCCTACC-3' and 5'-AGCCCAAGATGCCCTTCAGT-3'. The primer sequences of ERα and GAPDH were determined based on data from the GenBank in the National Institutes of Health, USA. The product length was 100 bp for ERα and 135 bp for GAPDH. The levels of ERα mRNA were calculated using a 2^-ΔΔCt ^method [25]. The method is based on the fact that the difference in threshold cycles (ΔCt) between the gene of interest (ERα) and housekeeping gene GAPDH is proportional to the relative expression level of the gene of interest.

### Statistical analysis

Significant differences in the quantitative data between the groups were analyzed using the Student's *t*-test via the method of Welch, which provides for insufficient homogeneity of variance. The differences in metastatic incidence were examined by Fisher's exact probability test, with *P *< 0.05 or *P *< 0.01 considered to represent a statistically significant difference.

## Results

### ER expression of mammary carcinoma cells

**Figure 1:**
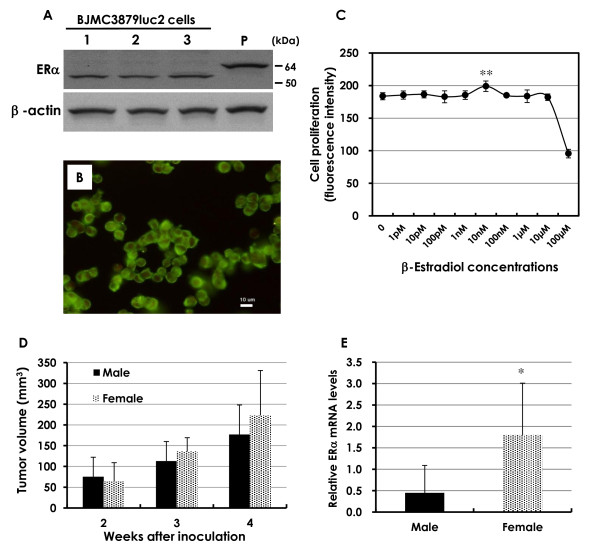
**ER expression of mammary carcinoma cells**. **A**. Western blots of ERα in mammary carcinoma BJMC3879luc2 cells showed bands between 50 and 64 kDa, but the MCF-7 human breast cancer cell line showed a ~66 kDa form. ERβ was not detected by western blots. **B**. In immunofluorescence staining, this smaller form showed cytoplasmic localization (green). Nuclear stain was conducted with PI (red). Bar = 10 μm. **C**. Cell proliferation was significantly increased by the addition of 10 nM E2 (**P < 0.01), but cell proliferation was not changed in any other concentration of E2, with the exception of the highest concentration of 100 μM, which is toxic. In the case of BJMC3879luc2-implanted tumors in mice, the tumor volume was slightly bigger in the female mice as compared to the male mice (**D**), and ERα mRNA levels were significantly higher in the implanted tumors in females than those in males (*P < 0.05) (**E**).

In the BJMC3879luc2 mammary carcinoma cell line, western blots showed ERα expression between 50 and 64 kDa, whereas the MCF-7 human breast cancer cell line expressed the 66-kDa form (Figure 1A). ERβ was not detected by western blots (data not shown). Immunofluorescence staining demonstrated that this smaller form of ERα was localized to the cytoplasm (Figure 1B). Cell proliferation was significantly increased by the addition of 10 nM of E2. However, other E2 concentrations did not change cell proliferation, with the exception of the highest concentration (100 μM), which was cytotoxic (Figure 1C). When tumor cells were implanted into female mice and male mice, the resulting tumor volume in the female mice was slightly larger, but the difference was not statistically significant (Figure 1D). However, the levels of ERα mRNA in the mammary tumors were significantly elevated in the females as compared to the males (Figure 1E).

### *In vitro *raloxifene study

#### Cell viability

**Figure 2:**
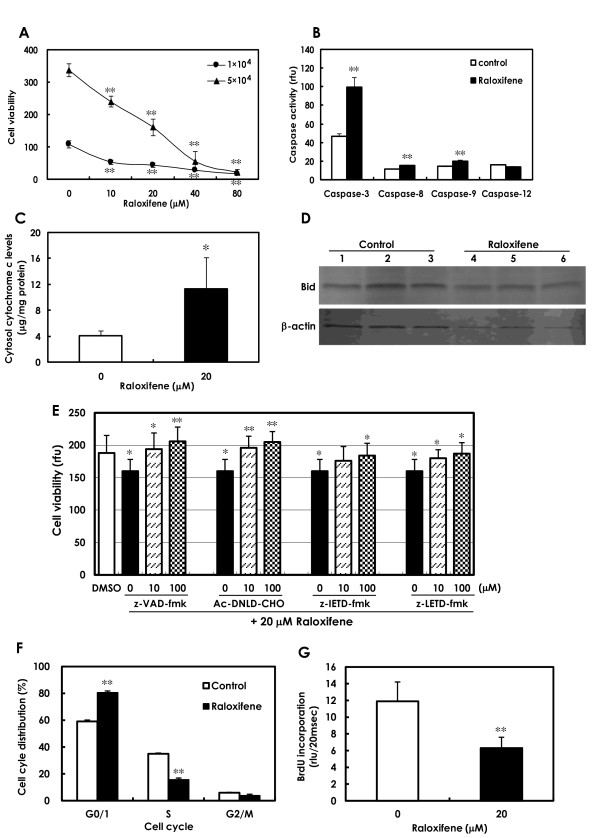
***In vitro *raloxifene study**. **A**. Cell viability was significantly decreased in mouse mammary carcinoma BJMC3879luc2 cells (1 × 10^4 ^cells and 5 × 10^4 ^cells/well) treated with more than 10 μM of raloxifene for 48 h (**P < 0.01). The IC50 concentration was determined to be 20 μM; therefore, 20 μM raloxifene for 48 h-incubation was used for *in vitro *studies. Ten samples from each dosage of raloxifene were examined. **B**. Caspase activities were evaluated according to luminescent assay. Activities of caspase-3, caspase-8 and caspase-9 (but not caspase-12) were significantly elevated in BJMC3879luc2 cells treated with 20 μM raloxifene for 48 h (**P < 0.01). Three samples each of control and raloxifene-treated cells were examined. **C**. Cytochrome *c *in the cytosolic fraction, as determined by ELISA, was significantly increased in cells treated with raloxifene for 48 h as compared to the control levels (*P < 0.05). Six samples from control cells and five samples from raloxifene-treated cells were examined. **D**. Western blots of Bid (22 kDa) in control cells and cells treated with raloxifene for 48 h were similar (upper panel). Cleaved Bid was not observed after raloxifene treatment. β-Actin served as an internal control (lower panel). **E**. In BJMC3879luc2 cells treated with raloxifene for 48 h, cell viabilities were significantly increased by the broad-spectrum caspase inhibitor z-VAD-fmk, the caspase-3 specific inhibitor Ac-DNLD-CHO, the caspase-8 specific inhibitor z-IETD-fmk, and the caspase-9 specific inhibitor z-LETD-fmk at 10 or 100 μM (*P < 0.05; **P < 0.01). Six samples each of control and raloxifene-treated cells were examined. **F**. Cell-cycle analysis showed that raloxifene induced arrest in the G1-phase and inhibition of the S-phase in metastatic mouse mammary carcinoma BJMC3879luc2 cells (**P < 0.01). Three samples each of control and raloxifene-treated cells were examined. **G**. Levels of DNA synthesis, as assessed by BrdU incorporation rates, were significantly decreased in the cells treated with raloxifene for 48 h (**P < 0.01). rlu: relative luminescent unit. Data presented are means ± SD values. Four samples each of control and raloxifene-treated cells were examined.

Cell viability of BJMC3879luc2 mammary cancer cells was significantly decreased after 48 h of treatment with more than 10 μM raloxifene (Figure 2A). The concentration of raloxifene in the *in vitro *study was determined to be 20 μM based on cell growth in the IC50 concentration. BJMC3879luc2 cells treated with 20 μM raloxifene for 48 h showed a greater number of apoptotic cells by TUNEL staining as compared to control (data not shown).

#### Caspase activities

Significantly elevated activities of caspase-3, caspase-8 and caspase-9 were observed in BJMC3879luc2 cells treated with raloxifene for 24 h (Figure 2B) and 48 h (data not shown), as compared to the respective controls. However, the activities of caspase-12 did not show significant differences between control cells and raloxifene-treated cells (Figure 2B).

#### Release of cytochrome c

Cytochrome *c *protein levels in cytosolic fractions were significantly elevated in cells treated with raloxifene for 48 h (Figure 2C). These findings strongly suggest the engagement of the mitochondria-mediated apoptotic pathway.

#### Bid cleavage

Since caspase-8 activities were elevated, we examined whether caspase-8-Bid cleavage via the mitochondrial pathway occurred by performing western blots for Bid. Full-length Bid (22 kDa) was detected in control cells and in cells treated with raloxifene for 48 h (Figure 2D). No cleaved Bid was found.

#### Caspase inhibitor experiment

To determine whether caspase activation is necessary to induce raloxifene-induced apoptosis, a caspase inhibitor experiment was conducted. The recovery of cell viability occurred in cells treated with all caspase inhibitors and raloxifene as compared with raloxifene alone for 48 h (Figure 2E).

#### Cell cycle and DNA synthesis

As measured by flow cytometry, 48 h exposure to 20 μM raloxifene induced a significant elevation in the numbers of cells in the G1-phase as compared with control cells (Figure 2F). There was also a significant reduction in the S-phase population in raloxifene-treated cell suspensions (Figure 2F). DNA synthesis in BJMC3879luc2 cells treated with raloxifene for 48 h, as assessed by BrdU incorporation, was significantly decreased (Figure 2G).

### *In vivo *raloxifene study

#### Body weights and mammary tumor growth

**Figure 3:**
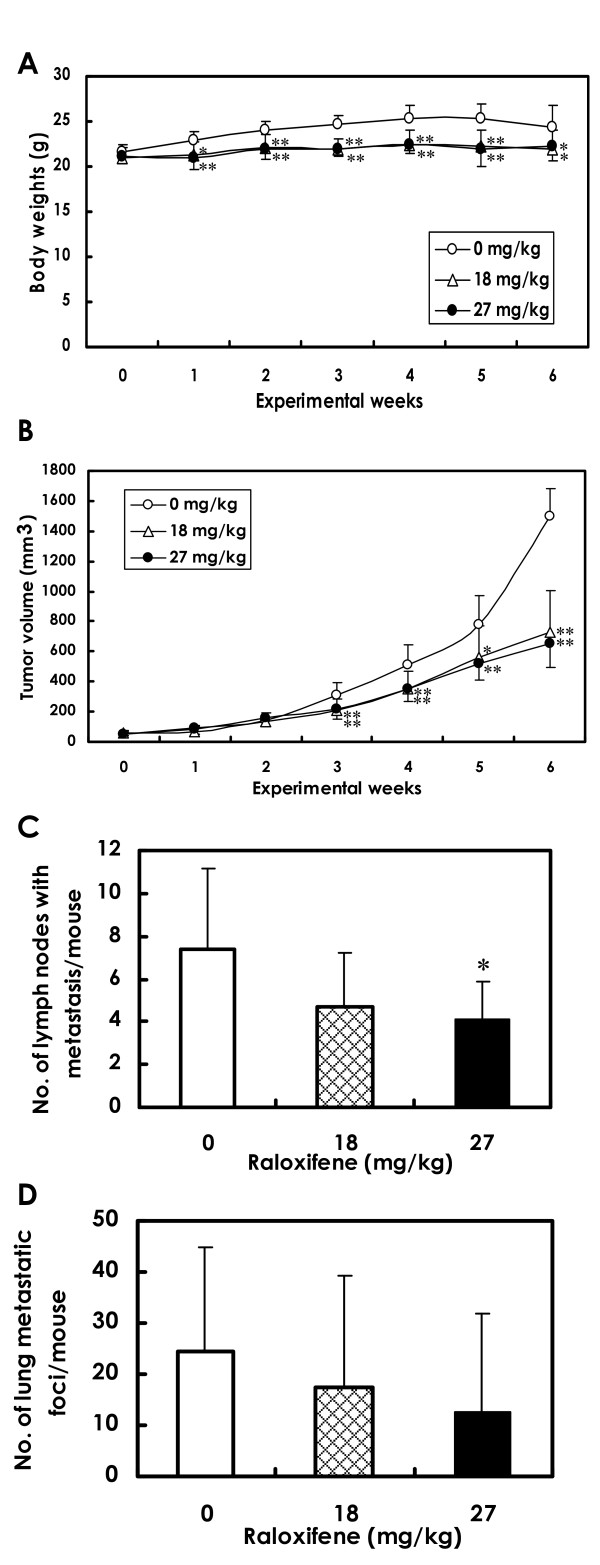
**Body weights, tumor volumes and multiplicity of metastasis in mammary carcinomas**. Raloxifene was administered with mini-osmotic pumps. Each group consisted of 10 mice. **A**. Body weights of mice treated with 18 and 27 mg/kg/day raloxifene were significantly decreased throughout the experiment as compared with the control group, but only by 10% (*P < 0.05; **P < 0.01). **B**. Tumor volumes in the 18 and 27 mg/kg/day groups began to decrease significantly as compared to the control values starting at week 3, and the differences became even more pronounced by the termination of the experiment (week 6) (*P < 0.05; **P < 0.01). **C**. Multiplicity of lymph node metastasis was significantly decreased in the 27 mg/kg raloxifene group (*P < 0.05). **D**. Multiplicity of lung metastasis tended to be reduced in the 27 mg/kg raloxifene group. Data are presented as means ± SD.

Body weight changes in control and raloxifene-treated mice bearing mammary tumors are shown in Figure 3A. The weights of mice treated with raloxifene (18 mg/kg or 27 mg/kg) were significantly lower than those of control mice throughout of the experiment. At the end of the study, the weight differences between the control group and the raloxifene-treated animals were 8~10%. One mouse from each group died at week 6 due to the mammary cancer metastasis. One mouse from the 27 mg/kg group died accidentally from an overdose of anesthesia when the osmotic mini-pumps were changed.

Tumor volumes are presented in Figure 3B. Tumor growth, as inferred by computed volume, was significantly inhibited in the 18 and 27 mg/kg groups from week 3 to the end of the experiment when compared with controls. By the end of the experiment, the average tumor volume in control animals was 1500 ± 183 mm^3^, while the average tumor volume of mice that received raloxifene was 729 ± 277 mm^3 ^(18 mg/kg) and 654 ± 161 mm^3 ^(27 mg/kg).

#### Metastasis of mammary carcinomas

**Figure 4:**
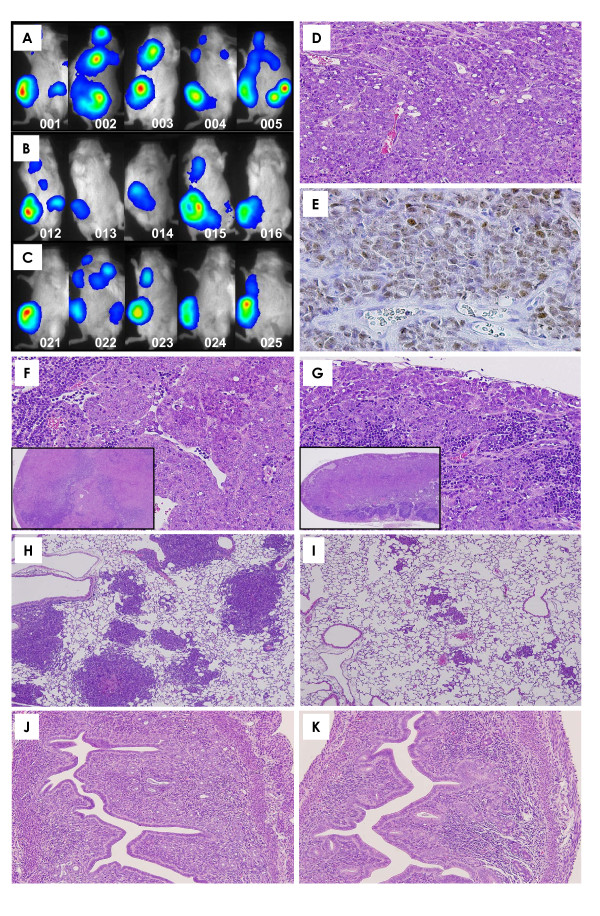
**Bioluminescent imaging and histopathological findings**. Bioluminescent imaging in five representative mice from each group (**A**, control; **B**, 18 mg/kg; **C**, 27 mg/kg). Bioluminescent imaging showed a tendency for decreases in the extension of metastasis in raloxifene-treated groups as compared to the control group. **D**. The implanted mammary carcinomas proved to be moderately differentiated adenocarcinoma. *×200*. **E**. p53 immunohistochemistry of mammary carcinoma induced by BJMC3879 cell inoculation. Note the nuclear staining for abnormal p53 protein, indicating that these cells carry mutant p53. *× 400*. **F**. Metastasis to lymph node in control mice *(×40*, inset). Metastatic carcinoma cells were filled with sinusoidal space (*× 400*). **G**. A lymph node from a mouse given 27 mg/kg raloxifene (*× 40*, inset). Metastatic carcinoma cells were filled with subcapsular sinus and sinusoidal space (*× 400*). **H**. Metastatic foci in the lung of a control mouse. Many metastatic foci and small to large nodules were seen. *× 40*. **I**. Metastatic foci in the lungs of mice given 27 mg/kg raloxifene. Metastatic lung foci were much smaller in the 27 mg/kg group than in the control group. *× 40*. **J **and **K**. Uterine endometrium was histopathologically similar between control and raloxifene-treated mice. *× 100*. **D **and **F-K**, *H&E stain*; **B**, *p53 immunohistochemistry*.

Bioluminescence imaging showed a tendency for metastatic expansion to be decreased in mice treated with raloxifene (Figure 4B, C) as compared to control animals (Figure 4A).

Histopathologically, the mammary carcinomas induced by BJMC3879luc2 cell inoculation proved to be moderately differentiated adenocarcinomas (Figure 4D) that contained mutated p53 as inferred by immunohistochemistry (Figure 4E).

### Lymph node metastasis

Representative lymph node metastases are shown in Figures 4F and 4G. Lymph node metastasis occurred in all mice independent of groups. However, the number of metastasis-positive lymph nodes per mouse was significantly decreased in the 27 mg/kg group as compared to the control group (Figure 3C).

### Lung metastasis

Lung metastasis occurred in all mice. The number of lung metastatic foci (>200 μm) per mouse tended to decreases in the raloxifene-treated groups, although the decrease was not statistically significant (Figure 3D). However, the metastatic foci tended to be smaller in the raloxifene-treated groups (Figure 4I) than in the control animals (Figure 4H). In addition, there was no difference in the uterine endometrium of the control mice and the raloxifene-treated mice (Figure 4J, K).

#### Apoptosis and DNA synthesis in mammary cancers

**Figure 5:**
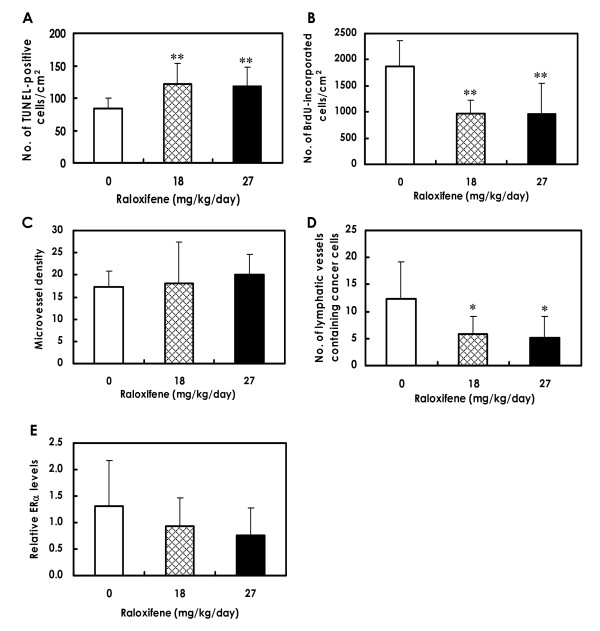
**Quantitative analyses of apoptosis, cell proliferation, vascular density and ERα expression in mammary carcinomas**. **A**. Apoptotic cell death, assessed by TUNEL assay, was significantly increased in the 18 and 27 mg/kg raloxifene groups (**P < 0.01). **B**. DNA synthesis, inferred by BrdU labeling indices, was significantly decreased in the 18 and 27 mg/kg raloxifene groups (**P < 0.01). **C**. Microvessel density in tumors, inferred by CD31-positive endothelium, was similar between the control group and the raloxifene-treated groups. **D**. The number of dilated lymphatic vessels containing intraluminal tumor cells was significantly lower in groups receiving 18 and 27 mg/kg raloxifene than in the control group (*P < 0.05). **E**. Levels of the truncated ERα mRNA tended to be decreased in the raloxifene-treated groups as compared to the levels of the control groups, but this difference was not significant as measured by real-time RT-PCR. Data are presented as means ± SD.

**Figure 6:**
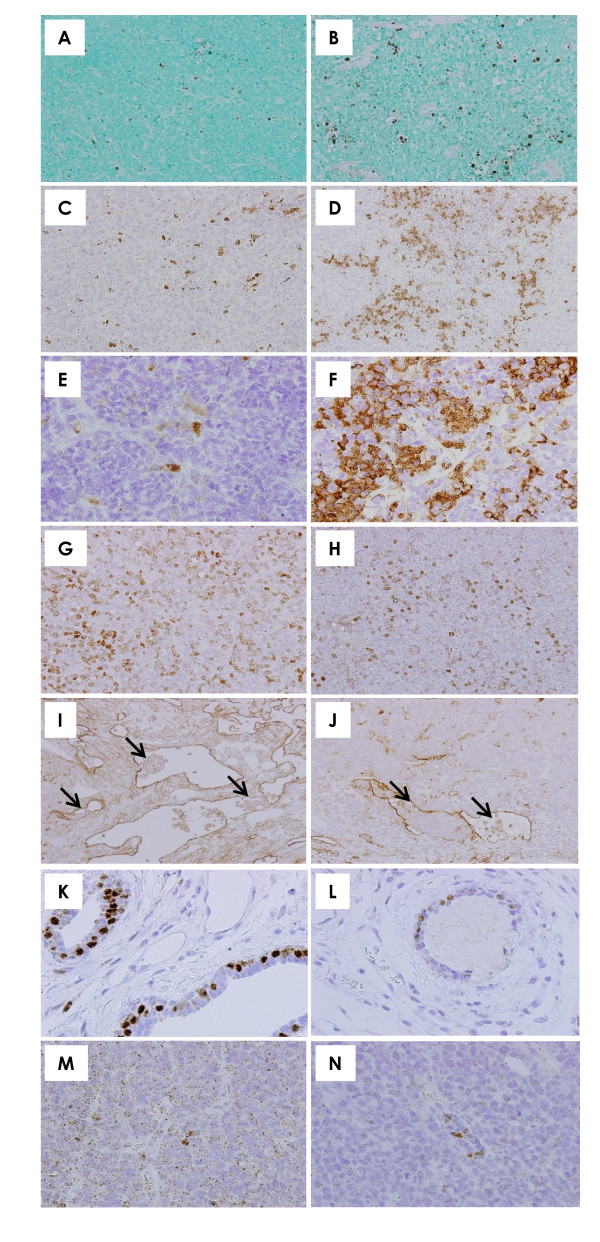
**Apoptosis, cell proliferation, lymphatic vessels with intraluminal tumor cells and ERα expression in mammary carcinomas**. Whereas some TUNEL-positive cells are seen in the tumor of a control mouse (**A**), many more TUNEL-positive cells are observed in the tumor of a mouse given 27 mg/kg raloxifene (**B**). *×200*. Expression of active caspase-3 (**C** and **D**, x200)and caspase-9 (**E** and **F**, x400) was more prominent in the tumor of a mouse given 27 mg/kg raloxifene (**D** and **F**) than in a control mouse (C and E). The number of BrdU-labeled cells tended to be lower in the 27 mg/kg raloxifene group (**H**) than in the control group (**G**). *×200*. Podoplanin-positive lymphatic vessels of a tumor in a control mouse were often dilated and filled with tumor cells (arrows, **I**). Raloxifene-treated groups showed a significant reduction in the numbers of dilated lymphatic vessels containing intraluminal tumor cells. (arrows, **J**). *×200*. The nuclear ERα expression of adjacent normal mammary glands in the mammary tumor was much stronger in the control (**K**) than in the 27 mg/kg raloxifene group (**L**). *×400*. The scattered expression of cytoplasmic ERα was much stronger in the control group (**M**) than in the 27 mg/kg raloxifene group (**N**). *×400*. **A **and **B**, *TUNEL stain; ***C **and **D**, *active caspase-3 immunohistochemistry*; **E **and **F**, *active caspase-9 immunohistochemistry*; **G **and **H**, *BrdU immunohistochemistry*; **I **and **J**, *podoplanin immunohistochemistry*. **K-N**, *ERα immunohistochemistry.*

Results of the quantitative analysis for apoptosis in lesions, as assessed by the TUNEL assay, are shown in Figure 5A. The number of TUNEL-positive cells was significantly increased in tumors from the 18 and 27 mg/kg groups (Figure 6B) as compared to the tumors from control mice (Figure 6A). Immunohistochemistry demonstrated that the expression of the active forms of caspase-3 and caspase-9 were much higher in mammary tumors treated with raloxifene (Figure 6D, F) than in the untreated control tumors (Figure 6C, E), suggesting that mitochondria-mediated apoptosis occurred in mammary tumor tissues exposed to raloxifene *in vivo*, too.

DNA synthesis levels in mammary carcinomas of raloxifene-treated mice (18 and 27 mg/kg), as inferred by BrdU labeling indices, are shown in Figure 5B. Levels of DNA synthesis in tumors were significantly decreased in the 18 and 27 mg/kg groups (Figure 5B and 6G, H).

#### Blood microvascular density and lymphatic vessels in mammary cancers

Microvessel density, as determined by immunohistochemical analysis with the blood vessel endothelial cell marker CD31, showed no statistically significant difference between control mice and raloxifene-treated mice (Figure 5C).

The lymphatic vessels in mammary tumors were stained with anti-podoplanin antibody, as demonstrated in Figures 6I and 6J. There were tumor cells within the lumina of dilated lymphatic vessels of tumors in both control (Figure 6I) and raloxifene-treated animals (Figure 6J). However, the number of dilated lymphatic vessels containing intraluminal tumor cells (arrows in Figure 6I, J) was significantly reduced in mammary tumors of mice given 18 and 27 mg/kg of raloxifene (Figure 5D), indicating a reduction in the number of tumor cells migrating into the lymphatic vessels of tumor tissues.

#### ERα expression of mammary carcinoma

Immunohistochemically, adjacent normal mammary glands in the mammary carcinomas showed nuclear expression of ERα in the control mice (Figure 6K), while the ERα expression level of normal mammary glands in the raloxifene-treated mice tended to be decreased (Figure 6L). In mammary carcinoma tissues, the scattered expression of ERα was observed in the cytoplasm of both control mice (Figure 6M) and raloxifene-treated mice (Figure 6N), and the expression level tended to decrease in the raloxifene-treated mice. In the quantitative analysis, levels of ERα mRNA in mammary carcinoma tissues tended to decrease in mice treated with raloxifene as compared to the control mice, but the decrease was not statistically significant (Figure 5E).

## Discussion

The present study showed that raloxifene inhibited tumor growth and multiplicity of metastasis to lymph nodes in a mouse immunocompetent metastatic mammary carcinoma model expressing cytoplasmic ERα. In addition, tumor tissues from the raloxifene-treated mice showed elevation of apoptotic cell death, suppression of DNA synthesis and inhibition of lymphatic vessels containing intraluminal cancer cells.

The present *in vitro *studies showed that the ERα expressed in the mammary carcinoma BJMC3879luc2 cells used in this study was between 50 and 64 kDa, which is smaller than the 66-kDa size of normal ERα, and it showed a cytoplasmic location. Cell proliferation of BJMC3879 cells expressing the smaller molecular weight ERα was significantly increased, but only by 7%, when added to 10 nM estrogen. When BJMC3879luc2 cells were implanted into mice, the ERα mRNA levels in the resultant tumors were significantly higher in female mice as compared to the male mice. Thus, although the ERα in the present study might be functional but weak, further investigation is necessary to elucidate this point. Recently, a truncated variant of 36-kDa ERα has been identified [26]. This subtype, which is predominantly localized to the cytoplasm and plasma membrane, responds to estrogen and mediates a nongenomic signaling pathway. Although this 36-kDa variant of ERα is apparently different from the present ERα, they share similarities in regard to localization and estrogen response.

The results of STAR [9], MORE [10], CORE [11] and RUTH [12] clinical trials show that raloxifene does not reduce the risk of ER-negative invasive breast cancer. Therefore, the fact that raloxifene exerted antimetastatic effects on mammary cancer expressing the cytoplasmic form of ERα may be an important finding with clinical applications. The question was raised as to why raloxifene exerted antitumor effects on mammary tumors that expressed the cytoplasmically located ERα in the present study. ER lacks known functional motifs that would allow for nongenomic mechanisms of estrogen action [27]. Raloxifene acts on both nuclear ERα and cytoplasmic ERα (nongenomic action) [28]. In this case, raloxifene does not target the estrogen response element; rather, it targets the raloxifene response element [29]. It was previously reported that estrogen activates cell proliferation in even ER-negative human breast cancer cells MDA-MB231 via GPR30, a member of the G protein-coupled receptor superfamily [30]. Thus, raloxifene can act by nongenomic mechanisms independent of ER, indicating the complexity and variety of SERMs. The biological effects of raloxifene decrease the ER levels [8,31]. In fact, in the present *in vivo *study, the mRNA levels of the truncated ERα in mammary tumors of raloxifene-treated mice showed a tendency to be decreased as compared to the levels in control mice. It is possible that raloxifene acts on the present mammary cancer model. In addition, ERα and β have been previously localized to mitochondria in various tissues [32,33]. In the present study, immunohistochemical localization of the truncated ERα revealed scattered expression in the cytoplasm, suggesting mitochondrial localization.

The present study demonstrated that raloxifene significantly induced apoptosis in murine mammary carcinoma cells both *in vitro *and *in vivo*. There are two pathways currently proposed to play major roles in regulating apoptosis in mammalian cells: a pathway mediated by death receptor (extrinsic pathway; execution by caspase-8) and a pathway mediated by mitochondria (intrinsic pathway; execution by caspase-9) [34]. Caspase-3 is a final executor of apoptosis. Many of the apoptosis signals are transduced to the mitochondria and decrease the mitochondrial membrane potential, which leads to the release of cytochrome *c *from the mitochondrial lumen into the cytoplasm. The released cytochrome *c *binds to the apoptosis protease-activating factor-1 (Apaf-1), and this complex activates caspase-9. Caspase-8 also has a cross-talk pathway to the mitochondria pathway through the cleavage of Bid [34].

In the present *in vitro *study, increases in caspase activities (caspase-3, -8 and -9) and cytosolic cytochrome *c *levels were found in mammary carcinoma cells treated with raloxifene, suggesting that raloxifene at least induced mitochondria-mediated apoptosis. Indeed, mammary cancer tissues of mice treated with raloxifene showed strong expression of active caspase-3 and -9 (cleaved forms), demonstrating that mitochondria-mediated apoptosis also occurred *in vivo*. All caspase inhibitors involving a caspase-8 inhibitor completely rescued raloxifene-induced cell death. However, since Bid cleavage was not observed, cross-talk between caspase-8 and Bid may not be involved. The question was raised as to why caspase-8 activity increased. Caspase-8 participates in ERK activation, and this regulation is attributed to the Death Effector Domains (DED) of caspase-8 [35]. Furthermore, a direct association between ERK and a DED-containing fragment of caspase-8, and co-transport of an ERK-caspase-8-DED complex to the nucleus during apoptosis has been reported [36]. The caspase-8-ERK pathway may also play a role in raloxifene-induced apoptosis. Further investigation is required to elucidate this point. In addition, caspase-12 mediates the pathway for cell death induced by endoplasmic reticulum stress in mice [37]. In the present study, since no elevation in caspase-12 activity was seen, the raloxifene-induced apoptosis may not have involved endoplasmic reticulum stress.

In animal carcinogenesis models, raloxifene at 20 mg/kg/day inhibits the tumor growth of 7, 12-dimethylbenzanthracene-induced mammary carcinomas in rats [5]. In mice, orally administered raloxifene (1.5 mg/mouse) reduces the tumor growth of mammary and endometrial cancer [23]. On the assumption that mouse body weights are 30 g, the dosage of raloxifene is estimated to be 50 mg/kg/day in mice. In carcinogenicity studies in mice and rats, raloxifene (8.7~225 mg/kg/day in mice; 10.4~259 mg/kg/day in rats) is not carcinogenic (company data from Eli Lilly Pharmaceuticals, Indianapolis, IN, USA). Although the clinical dosages of raloxifene in trials are 60 mg or 120 mg/day, a much higher dose of 600 mg/day (estimated as 10 mg/kg/day on the assumption that body weight is 60 kg) has also been used in clinical studies without adverse side effects [38,39]. Therefore, the doses of raloxifene used in the present mouse study (18 and 27 mg/kg/day) are not extremely high, and the dosage levels are considered to be near the clinical dose. However, low doses of raloxifene also exert antitumorigenic effects in animal cancer models [8].

Cancer cells metastasize to distal sites via the lymphatic system and the vascular system. The lymphatic capillaries present in tissues and tumors provide entrance into the lymphatics, allowing cancer cell migration to the lymph nodes. In the present study, it was demonstrated that the multiplicity of lymph node metastases was decreased in raloxifene-treated mice. This phenomenon was supported by a significant decrease in the number of lymphatic vessels with tumor cells in their lumina in the raloxifene-treated groups. This finding indicates that raloxifene may have an inhibitory effect on migration into lymphatic vessels. In fact, raloxifene has been reported to inhibit estrogen-induced cell migration and invasion through a non-nuclear signaling cascade involving G proteins and the RhoA-associated kinase [40]. It was also reported that raloxifene decreases levels of cyclooxygenase-2 and inducible nitric oxide synthase in carrageenan-induced inflammation of rats [41]. This mechanism could possibly be involved in the antitumorigenic effects of raloxifene.

Neovascularization is also a key process in the growth of solid tumors, and the growth of both primary tumors and metastases is thus angiogenesis-dependent [42]. However, in the present study, microvessel density in tumors was similar between the control and raloxifene-treated groups, indicating that raloxifene may not have anti-angiogenic action. However, the microvessel density in the 27 mg/kg raloxifene group was slightly increased. Since raloxifene induces cell proliferation and up-regulation of telomerase activity in human umbilical vein endothelial cells [43], this effect might be involved in the present study. However, since raloxifene did not inhibit angiogenesis in tumors in the present study, lung metastasis may not have been strongly inhibited.

The present experiments suggest that raloxifene-induced apoptosis in BJMC3879Luc2 cells having a p53 mutation occurs through a p53-independent mechanism. Since 50% of human cancers have *p53 *mutations [44], the fact that the raloxifene induces a p53-independent apoptotic response in cancer cells having a *p53 *mutation may be highly relevant to inhibiting many human cancers. In the case of non-functional p53 status, p73, the p53 homologue, may play a role in apoptosis induction.

## Conclusion

Our results demonstrated that treatment with raloxifene significantly suppresses lymph node metastasis in a mouse mammary cancer model expressing cytoplasmic ERα. The antimetastatic activity of raloxifene may be a crucial finding with clinical applications, and raloxifene may be useful as an adjuvant therapy and for the chemoprevention of breast cancer development.

## Abbreviations

BrdU: 5'-bromo-2'-deoxyuridine; CORE: Continuing Outcomes Relevant to Evista; DMSO: dimethylsulfoxide; E2: 17-β estradiol; GAPDH: glyceraldehyde-3-phosphate dehydrogenase; LSAB: labeled streptavidin-biotin; MORE: Results of other clinical trials of raloxifene, such as the Multiple Outcomes of Raloxifene Evaluation; MMTV: mouse mammary tumor virus; PBS: Phosphate-buffered saline; RT-PCR: reverse transcriptase-polymerase chain reaction; RUTH: Raloxifene Use for The Heart; SERM: selective estrogen receptor modulator; STAR: Study of Tamoxifen and Raloxifene; TUNEL: terminal deoxynucleotidyl transferase-mediated dUTP-FITC nick end-labeling.

## Competing interests

The authors declare that they have no competing interests.

## Authors' contributions

MAS performed the cell culture, animal experiments, Western blots, histopathology and statistical analysis. All *in vitro *studies (except for Western blots, cell-cycle analysis and ERα immunofluorescence) were performed by ES. Transplantation was performed by JM. Cell-cycle analysis was performed by HK. ERα immunofluorescence was conducted by KA. Experiments were performed by MAS, and ZL, MK, MO and YO participated in the design of the study. MAS wrote the manuscript. All authors have read and approved the final manuscript to be submitted.

## Pre-publication history

The pre-publication history for this paper can be accessed here:

http://www.biomedcentral.com/1471-2407/10/566/prepub
